# Effectiveness and Safety of Direct Oral Anticoagulant for Secondary Prevention in Asians with Atrial Fibrillation

**DOI:** 10.3390/jcm8122228

**Published:** 2019-12-17

**Authors:** Jiesuck Park, So-Ryoung Lee, Eue-Keun Choi, Soonil Kwon, Jin-Hyung Jung, Kyung-Do Han, Myung-Jin Cha, Sang-Bae Ko, Seil Oh, Gregory Y. H. Lip

**Affiliations:** 1 Division of Cardiology, Department of Internal Medicine, Seoul National University Hospital, Seoul 03080, Korea; 2 Department of Medical Statistics, College of Medicine, Catholic University of Korea, Seoul 06591, Korea; 3 Department of Neurology, Seoul National University Hospital, Seoul 03080, Korea; 4 Liverpool Centre for Cardiovascular Science, University of Liverpool and Liverpool Chest & Heart Hospital, Liverpool L14 3PE, UK; 5 Aalborg Thrombosis Research Unit, Department of Clinical Medicine, Aalborg University, 9000 Aalborg, Denmark

**Keywords:** atrial fibrillation, anticoagulants, stroke, secondary prevention

## Abstract

We investigated the effectiveness and safety of direct oral anticoagulants (DOACs) for secondary prevention in patients with atrial fibrillation (AF), particularly focusing on subgroups of patients with severe, disabling, and recent stroke. Using the Korean National Health Insurance Service claims database between January 2010 and April 2018, we selected OAC-naïve patients with non-valvular AF and a history of stroke. Cumulative risks for recurrent stroke, major bleeding, composite outcome (recurrent stroke + major bleeding), and mortality were compared between DOAC and warfarin groups. Among 61,568 patients, 28,839 and 32,729 received warfarin and DOACs, respectively. Compared with warfarin, DOACs were associated with lower risks of recurrent stroke (hazard ratio (HR) 0.67, 95% confidence interval (CI) 0.62–0.72), major bleeding (HR 0.73, 95% CI 0.66–0.80), composite outcome (HR 0.69, 95% CI 0.65–0.73), and mortality. DOAC use resulted in a consistent trend of improved outcomes in the subgroups of patients with severe, disabling, and recent stroke. In conclusion, DOACs were associated with lower risks of recurrent stroke, major bleeding, composite clinical outcomes, and mortality in patients with AF and a history of stroke. These results were consistent across all types of DOACs and subgroups of patients with severe, disabling, and recent stroke.

## 1. Introduction

Atrial fibrillation (AF) is a major risk factor for stroke. Compared to other causes, strokes caused by AF are usually more disabling and fatal [1]. Therefore, oral anticoagulation therapy (OAC) is essential for the prevention of stroke in patients with AF [2,3]. Among patients with AF, those with a history of stroke are categorized as a high-risk group, with a higher risk of recurrent stroke and bleeding events [4]; therefore, optimal antithrombotic therapy is imperative in these patients. 

Previous pivotal randomized clinical trials (RCTs) that investigated a varied number of patients with AF and a history of stroke reported that following subgroup analysis, the efficacy and safety profile of direct oral anticoagulants (DOACs) was comparable or better than that of warfarin [5,6,7,8]. However, most patients with severe, disabling, or recent stroke were excluded from the aforementioned RCTs; therefore, limited data are available in this high-risk population. A recent retrospective observational study reported that DOACs showed similar results with their respective RCT subgroup analysis in patients with a history of stroke or transient ischemic attack [9]. However, the results had a limitation because of the small sample size of the study. Moreover, there is a paucity of evidence regarding secondary prevention in Asian patients, who are reportedly at a higher risk of intracranial bleeding [10]. 

In this large nationwide population-based study, we investigated the effectiveness and safety of all four available DOACs (rivaroxaban, dabigatran, apixaban, and edoxaban) compared with that of warfarin in Asian patients with non-valvular AF and a history of stroke, particularly focusing on subgroups of patients with severe, disabling, and recent stroke.

## 2. Materials and Methods

Clinical data of the study population were obtained from the national health claims database established by the National Health Insurance Service (NHIS) of Korea. The NHIS is a mandatory universal health insurance service that provides comprehensive medical care to the entire Korean population [11,12]. This database includes claims data for medical services and medical costs across the Korean population and includes clinical information, such as demographics, diagnoses, examination details, as well as prescription and procedure records for inpatient and outpatient services. We used the International Classification of Disease, 10^th^ Revision, Clinical Modification (ICD-10-CM) codes for diagnostic classification of patients. This study was approved by the Institutional Review Board of Seoul National University Hospital (E-1808-022-963), and the informed consent was waived by the review board.

### 2.1. Study Design

Between January 2010 and April 2018, we identified patients with AF and a history of stroke who had received ≥1 prescription of any OAC (warfarin or DOACs (rivaroxaban, dabigatran, apixaban, or edoxaban)). The following were the exclusion criteria in this study: OAC treatment before 1 January 2010, valvular AF, end-stage renal disease, age <20 years, OACs indicated for conditions other than AF, including deep vein thrombosis, pulmonary embolism, or joint replacement surgery. Finally, we included 61,568 OAC-naive patients with non-valvular AF who received OACs (warfarin (28,839) and DOACs (32,729)) for secondary stroke prevention during the study period (Figure S1).

### 2.2. Covariates

Patient demographics, comorbidities, and details of concomitant medications were retrieved from the database. Table S1 summarizes the detailed definitions (based on ICD-10-CM codes) of comorbidities, including hypertension, diabetes mellitus, dyslipidemia, congestive heart failure, prior myocardial infarction, peripheral artery disease, renal disease, chronic obstructive pulmonary disease, cancer, and a history of intracranial hemorrhage (ICH) and gastrointestinal bleeding (GIB) [13,14,15]. The CHA_2_DS_2_-VASc score to evaluate stroke risk, the HAS-BLED score (Hypertension (1 point), liver disease (1 point), renal disease (1 point), stroke history (1 point), bleeding history (1 point), age >65 years (1 point) and drug (concomitant use of NSAID or antiplatelet agent, 1 point)) to assess bleeding risk (Table S1), and the Charlson comorbidity index (CCI) that quantifies a patient’s comorbidity burden was calculated by combining covariates [16,17,18]. We analyzed the records of concomitant prescriptions of antiplatelet agents (aspirin or clopidogrel), non-steroidal anti-inflammatory drugs, and proton pump inhibitors.

### 2.3. Study Outcomes and Follow-Up

The first date of index OAC prescription was defined as the index date of follow-up. Patients were censored at the occurrence of the outcome events, discontinuation of index OAC therapy, or at the end of the study period (30 April 2018), whichever occurred first. Discontinuation was defined as a lack of prescription records for OAC 30 days after the last date of index drug prescription. The study outcomes included recurrent stroke, major bleeding (ICH + hospitalization for GIB), composite outcome (recurrent stroke + major bleeding), death from each clinical outcome, and all-cause death. A fatal event was defined as any clinical outcome causing death during the index hospitalization. Detailed definitions of clinical outcomes are summarized in Table S1. 

### 2.4. Statistical Analyses

We compared the risk of clinical outcomes between (i) warfarin and pooled DOACs and (ii) warfarin and the individual DOACs using Kaplan–Meier survival analysis with log-rank test and weighted Cox proportional hazard regression model. Inverse probability of treatment weighting (IPTW) using propensity scores (PS) was performed to calculate the weights for each comparison [19]. All baseline covariates were subjected to ordinal logistic regression analysis to calculate the PS of being assigned to a treatment group [18]. We considered the maximum absolute standardized difference (ASD) of 0.1 (10%) as a negligible difference in baseline characteristics between the comparators [19,20]. After IPTW, incidence rates (IRs) were calculated for all clinical outcomes using weighted event numbers per 100 person-years. With regard to the absolute measures of clinical benefit, we estimated absolute risk reduction (ARR) at 1 year follow-up and the number needed to treat (NNT) to prevent one event for each clinical outcome with DOAC vs. warfarin use. Statistical analyses were performed using the SAS software, version 9.3 (SAS Institute Inc., Cary, NC, USA), and statistical significance was defined as *p* < 0.05. 

### 2.5. Subgroup and Sensitivity Analyses

The risk of clinical outcomes between the pooled DOAC and warfarin groups was compared across subgroups stratified by age, sex, and comorbidities using a multivariable-adjusted Cox proportional hazard model based on all covariates included for PS calculation. Additionally, we focused on subgroups of patients with severe, disabling, and recent stroke. Notably, these categories of patients have largely been excluded or are under-represented in large-scale RCTs. We used the following definitions for these subgroups: patients with severe stroke were those who required intensive care unit (ICU) admission for a history of stroke, patients with disabling stroke were those requiring rehabilitation therapy after stroke-induced disability, and patients with recent stroke were those with a stroke within 6 months before the index inclusion date.

Additionally, a weighted hazard model with IPTW was used to evaluate clinical outcomes only in patients receiving regular-dose DOACs (rivaroxaban 20 mg, dabigatran 150 mg, apixaban 5 mg, and edoxaban 60 mg) at index prescriptions. A significant percentage of Asian patients with AF are often prescribed reduced doses of DOACs without definitive indication [13,21]. Therefore, we concluded that sensitivity analysis restricted to patients receiving regular-dose DOACs would be useful to compare the different types of DOACs in real-world settings. Finally, a sensitivity analysis was performed with 1 year follow-up to equipoise the follow-up period between the DOAC and warfarin groups.

## 3. Results

### 3.1. Patients’ Baseline Characteristics

Among the 61,568 patients with non-valvular AF and a history of stroke who received OACs, 28,839 (46.8%), and 32,729 (53.2%) patients received warfarin and DOACs, respectively. Table 1 summarizes patients’ baseline characteristics. Compared with the warfarin group, patients in the DOAC group were older with a higher mean CHA_2_DS_2_-VASc score but lower mean HAS-BLED and CCI scores. The warfarin group showed a higher prevalence of concomitant use of antiplatelet agents but a lower prevalence of previous GIB. 

With regard to the types of DOACs administered, 12,311 (37.6%), 6293 (19.1%), 8837 (27.0%), and 5288 (16.2%) patients received rivaroxaban, dabigatran, apixaban, and edoxaban, respectively. All covariates were well balanced after IPTW (ASDs for all covariates ≤0.1) (Table S2; Figure S2) and the mean age was 74 years with a mean CHA_2_DS_2_-VASc score of 5.9.

### 3.2. Recurrent Stroke, Major Bleeding, Composite Outcome, Death from Each Clinical Outcome, and All-Cause Death 

We observed that during a median follow-up of 0.7 years (interquartile range 0.2–1.9 years), recurrent stroke, major bleeding, and composite outcome occurred in 5.7% (*n* = 3507), 3.1% (*n* = 1905), and 8.4% (*n* = 5189) of patients, respectively. Table 2 shows the weighted hazard ratios (HRs) for clinical outcomes after IPTW. Compared with warfarin, DOAC use was associated with a lower risk of recurrent stroke (HR 0.67, 95% confidence interval (CI) 0.62–0.72), major bleeding (HR 0.73, 95% CI 0.66–0.80), and composite outcome (HR 0.69, 95% CI 0.65–0.73). DOAC use was also associated with a lower risk of fatal recurrent stroke (HR 0.69, 95% CI 0.59–0.79), fatal major bleeding (HR 0.50, 95% CI 0.37–0.68), fatal composite outcome (HR 0.65, 95% CI 0.57–0.74), and all-cause death (HR 0.84, 95% CI 0.80–0.89). 

Figure S3 shows the weighted Kaplan–Meier curves for clinical outcomes in the DOAC and warfarin groups. DOAC use was associated with a better event-free survival rate with regard to all clinical outcomes.

### 3.3. Comparison between Warfarin and Individual Non-Vitamin K Oral Anticoagulants 

**Figure 1:**
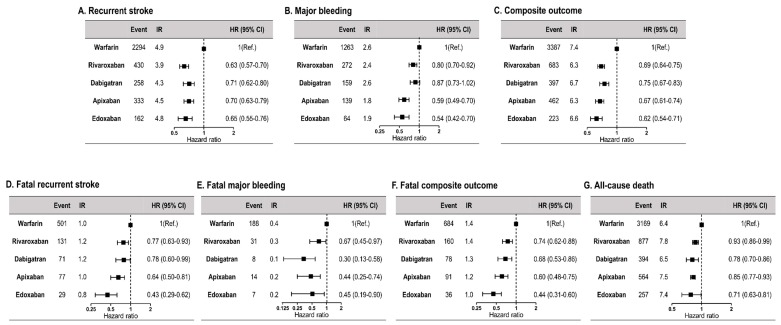
Comparison of clinical outcomes according to antithrombotic therapy (warfarin vs. four DOACs). CI, confidence interval; DOAC, direct oral anticoagulant; HR, hazard ratio; IR, incidence rate.

Figure 1 shows the weighted HRs for clinical outcomes in the warfarin and the four DOAC groups. Compared with warfarin, all four DOACs showed a lower risk of clinical outcomes. With regard to major bleeding and the composite outcome, compared to other DOACs, edoxaban showed the lowest risk (HR 0.54, 95% CI 0.42–0.70 for major bleeding and HR 0.62, 95% CI 0.54–0.71 for composite outcome). Compared to warfarin, all four DOACs were associated with a lower risk of death from each clinical outcome and all-cause death. Among the four DOACs, edoxaban showed the lowest risk of fatal recurrent stroke (HR 0.43, 95% CI 0.29–0.62), fatal composite outcome (HR 0.44, 95% CI 0.31–0.60), and all-cause death (HR 0.72, 95% CI 0.63–0.81). Weighted Kaplan–Meier curves for clinical outcomes of the four DOACs are shown in Figure S4.

### 3.4. The Number Needed to Treat for Clinical Outcomes in the Non-Vitamin K Oral Anticoagulant and Warfarin Groups

Table 3 shows the 1 year ARR and NNT values for (i) pooled DOACs and, (ii) each type of DOAC compared with warfarin. Among the pooled DOACs, the NNT values to prevent one recurrent stroke and one composite event were 65 and 48, respectively. The NNT for preventing one death with the use of DOACs was 94. 

Among the four types of DOACs, compared with warfarin, edoxaban showed the lowest NNT with regard to all clinical outcomes except major bleeding. The NNT to prevent one composite outcome event was 35 in patients receiving edoxaban.

### 3.5. Subgroup Analyses

The crude IRs of clinical outcomes among the subgroups are summarized in Table S3. Subgroup analysis for age, sex, baseline CHA_2_DS_2_-VASc, HAS-BLED, and CCI scores, diabetes mellitus, hypertension, chronic kidney disease, and heart failure were consistent with our main results, although significant interactions were observed with regard to hypertension (ischemic stroke and composite outcome), chronic kidney disease (composite outcome), and CCI (recurrent stroke, major bleeding, composite outcome, and all-cause death) (Figure S5). DOACs showed a consistent trend of risk reduction with regard to all clinical outcomes across all subgroups.

### 3.6. Severe, Disabling, and Recent Stroke 

**Figure 2:**
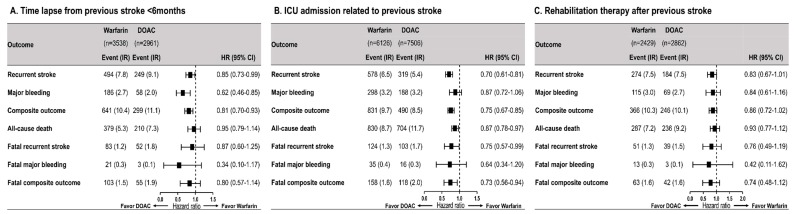
Comparison of clinical outcomes according to antithrombotic therapy among subgroups with recent or severe/disabling stroke. CI = confidence interval, DOAC = direct oral anticoagulant; HR = hazard ratio, ICU = intensive care unit, IR = incidence rate.

Figure 2 shows the HRs for clinical outcomes among subgroups of patients with recent stroke (*n* = 6499), patients with a prior stroke-related ICU admission (*n* = 13,632), and patients requiring rehabilitation therapy secondary to disabling stroke (*n* = 5291). Compared to warfarin, DOACs showed a consistent trend of risk reduction with regard to all clinical outcomes across all three subgroups.

### 3.7. Sensitivity Analysis

Sensitivity analysis using a multivariable-adjusted Cox proportional hazard model showed findings similar to our main results with regard to pooled DOACs (Table S4) and the four types of DOACs individually (Table S5). Among patients administered DOACs, 12,073 (36.9%) received regular-dose DOACs (Table S6). Compared with those administered warfarin, patients who received regular-dose DOACs showed significantly improved clinical outcomes (Table S7; Figure S6). Compared with warfarin, the clinical improvement observed with regular doses of the four types of DOACs was similar to our main results except with regard to fatal major bleeding because of the relatively small numbers of events (Figure S7; Figure S8). Among the four types of DOACs, edoxaban was associated with the lowest risk of most clinical outcomes. Sensitivity analysis with 1 year follow-up showed that the HRs for all clinical outcomes followed trends that resembled those observed in our main analysis (Figure S9).

## 4. Discussion

In this large nationwide population-based study, we investigated the effectiveness and safety of DOACs compared with that of warfarin for secondary prevention among Asian patients with non-valvular AF. This study highlights the following findings: (1) Compared to warfarin, DOAC use was associated with lower risks of recurrent stroke, major bleeding, and composite outcome. (2) DOAC use was also associated with lower risks of fatal recurrent stroke, fatal major bleeding, fatal composite outcome, and all-cause death. (3) Consistent trends in effectiveness and safety were observed across all four types of DOACs and regular-dose DOACs. (4) Following subgroup analysis, DOACs showed better clinical outcomes in patients with severe, disabling, and recent stroke.

To our knowledge, this is the largest study that investigated patients with AF and a history of stroke to compare the effectiveness and safety of DOACs with that of warfarin, particularly focusing on the aforementioned subgroups that have been under-represented in previous clinical trials.

A recent meta-analysis of four major clinical trials reported that compared to warfarin, DOACs showed a significantly lower risk of stroke or systemic embolism in patients with AF and a history of stroke [22]. Several studies using population-based claims databases have investigated the effectiveness and safety of different types of DOACs compared with that of warfarin in patients with AF and a history of stroke [9,23,24,25]. A Danish nationwide cohort study reported that the risks of ischemic stroke or systemic embolism associated with apixaban, dabigatran, and rivaroxaban were comparable to those associated with warfarin [23]. With regard to safety outcomes, dabigatran was associated with a lower risk of major bleeding, whereas the risk profile of apixaban and rivaroxaban was comparable with that of warfarin. Another study using the MarketScan claims data reported that rivaroxaban showed a lower risk of composite endpoint of ischemic stroke or ICH, whereas the risk profile of apixaban and dabigatran was comparable to that of warfarin [9]. Notably, both studies included a relatively small number of patients with AF and a history of stroke; therefore, these studies were of low statistical power (Danish study (*n* = 9463) and US MarketScan claims study (*n* = 9684)).

The clinical benefit of DOACs in risk reduction of fatal clinical outcomes (fatal recurrent stroke, fatal major bleeding, and death from composite outcome) observed in our study was consistent with that reported by previous RCTs [5,6,7,8]. We estimated the ARR and NNT for each clinical outcome associated with DOACs compared to warfarin to calculate the expected absolute benefit in individual patients. The NNT to prevent one recurrent stroke event with the use of DOACs was 65. With regard to safety outcomes, the NNT to prevent one major bleeding event with the use of DOACs was 142, and compared with warfarin, DOAC use resulted in a more favorable NNT value of 48 to prevent one composite outcome. Increasing numbers of patients with AF and a history of stroke are being encountered in clinical practice; therefore, our results emphasize the importance of DOAC use considering their cost-effectiveness for secondary stroke prevention in patients with AF [26].

A recent study using Taiwan’s National Health Insurance Research Database investigated patients with AF and a history of stroke and reported that all four types of DOACs were associated with a lower risk of ischemic stroke or systemic embolism; edoxaban use led to the highest risk reduction in these patients [24]. With regard to safety outcomes, all four types of DOACs showed similar efficacy in reducing the risk for major bleeding; however, edoxaban and apixaban were associated with greater risk reduction than dabigatran and rivaroxaban.

Previous pivotal RCTs excluded patients with AF and a history of stroke within 1–2 weeks preceding randomization and those with severe/disabling stroke. Therefore, data describing the clinical benefit of DOACs vs. warfarin in this population are limited. A strength of our study is the large-sized real-world cohort comprising heterogeneous subgroups that may have been under-represented in RCTs. Therefore, we performed subgroup analyses to determine whether the benefits of DOACs were consistent across all patient categories (those with recent stroke [<6 months] and those with severe and disabling stroke). We observed that compared to warfarin, DOACs showed similar trends in reducing risks of clinical outcomes across all subgroups, although the IR of the fatal clinical outcomes was relatively low. Our results support DOAC use in this patient population based on their proven clinical benefits.

The issue of appropriate dosing for DOACs in real-world clinical practice is particularly important where insufficient anticoagulation for stroke prevention is prevalent [21]. A recent meta-analysis reported that compared to warfarin, regular-dose DOACs showed a significantly lower risk of ischemic stroke with comparable risks of major bleeding for secondary prevention in cases of AF [27]. Several observational studies indicate that inappropriate prescriptions of low-dose DOACs with poor adherence to label- or guideline recommendations (particularly with apixaban) increase the risk of thromboembolic events without reducing bleeding events [24,25]. In the current study, approximately one-third (36.9%) of all patients received regular-dose DOACs and compared to warfarin, regular-dose DOACs were consistently associated with better clinical outcomes and lower risks of recurrent stroke, major bleeding, and composite outcome. In line with previous studies, our results indicate that it is important to prescribe the label- or guideline-recommended dose of DOACs for effective secondary stroke prevention in patients with AF in real-world practice.

### Study Limitations

The following are the limitations of this study that should be addressed. (1) This retrospective study utilized information obtained from a nationwide claims database. Although we used IPTW to adjust for baseline differences between treatment groups, there might be uncaptured residual confounders that could affect treatment effects. (2) Laboratory investigations, such as the international normalized ratio (based on the results of prothrombin time), are not included in the NHIS database; therefore, the quality of anticoagulation in the warfarin group could not be confirmed. Notably, the quality of anticoagulation in Korean patients with ischemic stroke and AF was shown to be low, with a mean time in therapeutic range of 49% [28]. Our results need careful interpretation considering inadequate anticoagulation in Asian patients receiving warfarin [29]. (3) We could not evaluate the actual drug adherence to OACs (warfarin and DOACs) in individual patients, which is an inherent limitation of any real-world study. (4) We investigated the clinical benefits of individual DOACs compared with those of warfarin; however, the current study was not focused on direct comparisons between the different types of DOACs. (5) The NHIS database does not contain data for body weight and creatinine clearance; therefore, we could not assess the appropriateness of DOAC dosing used for each patient. (6) Among the patients with DOACs, 63.1% of them received reduced dose DOACs which could inflate the clinical benefit of DOACs compared to warfarin. However, we performed additional sensitivity analysis restricted to patients who received regular dose DOACs and found a consistent trend for the effectiveness and safety of DOACs compared to warfarin even after excluding those with reduced dose DOACs.

## 5. Conclusions

DOACs were associated with significantly lower risks of recurrent stroke, major bleeding, composite clinical outcome, and mortality in patients with AF and a history of stroke. The results were consistent across different types and doses of DOACs and across subgroups of patients with recent, severe, and disabling stroke.

## Figures and Tables

**Table 1:** Baseline characteristics of the study population.

	Warfarin	DOAC	ASD	Rivaroxaban	Dabigatran	Apixaban	Edoxaban	ASD
	(*n* = 28,839)	(*n* = 32,729)		(*n* = 12,311)	(*n* = 6293)	(*n* = 8837)	(*n* = 5288)	
**Age**								
Mean ± SD, years	73.1 ± 9.4	75.2 ± 9.1	0.233	75.4 ± 8.8	73.9 ± 9.5	75.9 ± 9.1	75.4 ± 9.0	0.253
<65 years	4757 (16.5)	3974 (12.1)		1389 (11.3)	956 (15.2)	981 (11.1)	648 (12.3)	
65–74 years	10,255 (35.6)	9775 (29.9)		3717 (30.2)	2035 (32.3)	2462 (27.9)	1561 (29.5)	
75≤ years	13,827 (48.0)	18,980 (58.0)		7205 (58.5)	3302 (52.5)	5394 (61.0)	3079 (58.2)	
**Male**	15,338 (53.2)	16,703 (51.0)	0.043	6037 (49.0)	3453 (54.9)	4427 (50.1)	2786 (52.7)	0.083
**CHA_2_DS_2_-VASc score**								
Mean ± SD	5.8 ± 1.5	5.9 ± 1.4	0.123	6.0 ± 1.4	5.8 ± 1.5	6.02 ± 1.41	5.9 ± 1.42	0.143
**HAS-BLED score**								
Mean ± SD	4.2 ± 1.1	4.2 ± 1.1	0.029	4.3 ± 1.1	4.15 ± 1.1	4.24 ± 1.11	4.1 ± 1.11	0.020
≥3	27,534 (95.5)	30,997 (94.7)		11,715 (95.2)	5922 (94.1)	8417 (95.3)	4943 (93.5)	
**CCI**								
Mean ± SD	5.2 ± 2.5	4.8 ± 2.5	0.125	4.8 ± 2.5	4.8 ± 2.5	5.0 ± 2.5	4.5 ± 2.5	0.124
≥3	24,466 (84.8)	26,808 (81.9)		10,085 (81.9)	5176 (82.3)	7432 (84.1)	4115 (77.8)	
**Time lapse from previous stroke**								
Mean ± SD, years	1.5 ± 1.9	2.6 ± 2.8		2.7 ± 2.7	2.2 ± 2.6	2.6 ± 2.9	3.0 ± 3.0	
Median (IQR), years	0.6 (0.1–2.5)	1.4 (0.1–4.7)		1.7 (0.2–4.8)	0.9 (0.1–3.9)	1.2 (0.1–4.7)	2.0 (0.1–5.4)	
**Hypertension**	26,072 (90.4)	29,265 (89.4)	0.033	11,056 (89.8)	5578 (88.6)	7945 (89.9)	4686 (88.6)	0.020
**Diabetes mellitus**	9869 (34.2)	10,467 (32.0)	0.048	3885 (31.6)	1971 (31.3)	3036 (34.4)	1575 (29.8)	0.057
**Dyslipidemia**	18,774 (65.1)	21,899 (66.9)	0.038	7890 (64.1)	4484 (71.3)	6062 (68.6)	3463 (65.5)	0.021
**Congestive heart failure**	11,972 (41.5)	14,780 (45.2)	0.074	5389 (43.8)	2751 (43.7)	4175 (47.2)	2465 (46.6)	0.046
**Myocardial infarction**	1836 (6.4)	1829 (5.6)	0.033	705 (5.7)	325 (5.2)	553 (6.3)	246 (4.7)	0.027
**Peripheral arterial disease**	7668 (26.6)	9075 (27.7)	0.026	3510 (28.5)	1816 (28.9)	2269 (25.7)	1480 (28.0)	0.043
**COPD**	4080 (14.2)	3994 (12.2)	0.058	1620 (13.2)	687 (10.9)	1158 (13.1)	529(10)	0.029
**Cancer**	2000 (6.9)	2541 (7.8)	0.032	970 (7.9)	449 (7.1)	763 (8.6)	359 (6.8)	0.036
**Previous Intracranial hemorrhage**	1530 (5.3)	1959 (6.0)	0.029	719 (5.8)	404 (6.4)	548 (6.2)	288 (5.5)	0.023
**Previous gastrointestinal bleeding**	5039 (17.5)	7678 (23.5)	0.149	2878 (23.4)	1396 (22.2)	2117 (24.0)	1287 (24.3)	0.147
**Antiplatelets**			0.440					0.431
No antiplatelets	12,731 (44.2)	21,454 (65.6)		8016 (65.1)	4097 (65.1)	5705 (64.6)	3636 (68.8)	
Aspirin only	7990 (27.7)	5183 (15.8)		2006 (16.3)	1021 (16.2)	1410 (16.0)	746 (14.1)	
Clopidogrel only	2816 (9.8)	3209 (9.8)		1199 (9.7)	621 (9.9)	870 (9.8)	519 (9.8)	
Dual antiplatelets	5302 (18.4)	2883 (8.8)		1090 (8.9)	554 (8.8)	852 (9.6)	387 (7.3)	
**NSAID**	13,940 (48.3)	14,321 (43.8)	0.092	5799 (47.1)	2902 (46.1)	3617 (40.9)	2003 (37.9)	0.025
**Proton pump inhibitors**	9090 (31.5)	12,073 (36.9)	0.113	4429 (36.0)	2516 (40.0)	3336 (37.8)	1792 (33.9)	0.094

The numbers are presented as the mean ± standard deviation, median (IQR), or numbers (percentage) otherwise mentioned. Abbreviation: ASD, absolute standardized difference; CCI, charlson comorbidity index; COPD, chronic obstructive pulmonary disease; DOAC, direct oral anticoagulant; IQR, interquartile range; NSAID, non-steroidal anti-inflammatory drug; SD, standard deviation. HAS-BLED score: Hypertension (1 point), liver disease (1 point), renal disease (1 point), stroke history (1 point), bleeding history (1 point), age >65 years (1 point) and drug (concomitant use of NSAID or antiplatelet agent, 1 point).

**Table 2:** The cumulative risk of clinical outcomes according to antithrombotic therapy.

Outcome	Warfarin	DOAC	*HR (95% CI)	*p*-Value
	Event (IR)	Event (IR)		
**Recurrent stroke**	2294 (4.9)	1184 (4.2)	0.67 (0.62–0.72)	<0.001
**Major bleeding**	1263 (2.6)	633 (2.2)	0.73 (0.66–0.80)	<0.001
**Composite outcome (Recurrent stroke + major bleeding)**	3387 (7.4)	1765 (6.4)	0.69 (0.65–0.73)	<0.001
**Fatal recurrent stroke**	501 (1.0)	307 (1.1)	0.69 (0.59–0.79)	<0.001
**Fatal major bleeding**	188 (0.4)	60 (0.2)	0.50 (0.37–0.68)	<0.001
**Fatal composite outcome**	684 (1.4)	366 (1.3)	0.65 (0.57–0.74)	<0.001
**All-cause death**	3169 (6.4)	2092 (7.4)	0.84 (0.80–0.89)	<0.001

* Inverse probability of treatment weighting (IPTW) adjustment. Abbreviation: CI, confidence interval; DOAC, direct oral anticoagulant; HR, hazard ratio; IR, incidence rate.

**Table 3:** The 1 year ARR and NNT with pooled DOACs and each DOAC compared to warfarin.

Outcome	Pooled DOAC		Rivaroxaban		Dabigatran		Apixaban		Edoxaban	
	1 year ARR	NNT	1 year ARR	NNT	1 year ARR	NNT	1 year ARR	NNT	1 year ARR	NNT
**Recurrent stroke**	1.5	65	1.7	60	1.3	78	1.0	100	2.3	44
**Major bleeding**	0.7	142	0.4	284	0.8	119	1.0	98	0.7	135
**Composite outcome (Recurrent stroke + major bleeding)**	2.1	48	1.9	54	2.0	49	1.8	54	2.9	35
**All-cause death**	1.1	94	0.6	180	1.3	77	0.6	179	2.7	38
**Fatal composite outcome**	0.5	190	0.6	175	0.4	271	0.3	287	0.9	118

Abbreviation: ARR, absolute risk reduction; DOAC, direct oral anticoagulant; NNT, the number needed to treat.

## Supplementary Materials

The following are available online at https://www.mdpi.com/2077-0383/8/12/2228/s1, Table S1. Definition of covariates and study outcomes. Table S2. Characteristics of study population after IPTW adjustment. Table S3. Subgroup analysis of clinical outcomes according to antithrombotic therapy. Table S4. Crude and multivariate-adjusted risk of clinical outcomes according to antithrombotic therapy. Table S5. Crude and multivariate-adjusted risk of clinical outcomes according to antithrombotic therapy (warfarin vs. four DOACs). Table S6. Characteristics of the study population with warfarin and dosage of DOAC. Table S7. Comparison of clinical outcomes between patients with warfarin and regular dose DOAC. Figure S1. Study flow. Figure S2. Distribution of propensity scores before and after IPTW. Figure S3. Kaplan–Meier curve of clinical outcomes according to antithrombotic therapy (warfarin vs. pooled DOACs). Figure S4. Kaplan–Meier curve of clinical outcomes according to antithrombotic therapy (warfarin vs. four DOACs). Figure S5. Subgroup analysis of clinical outcomes according to antithrombotic therapy. Figure S6. Kaplan–Meier curve of clinical outcomes among patients with warfarin and regular dose DOAC. Figure S7. Comparison of clinical outcomes between patients with warfarin and four regular dose DOACs. Figure S8. Kaplan–Meier curve of clinical outcomes among patients with warfarin and four different regular dose DOAC. Figure S9. Sensitivity analysis comparing clinical outcomes according to antithrombotic therapy during a 1 year follow-up.
