# Supplementary material for: Effectiveness and Safety of Direct Oral Anticoagulant for Secondary Prevention in Asians with Atrial Fibrillation
Source: J Clin Med. 2019 Dec 17;8(12):2228. doi: 10.3390/jcm8122228 (PMC6947173; doi:10.3390/jcm8122228)
Supplement: Supplementary file 1 [file jcm-08-02228-s001.pdf]

Supplementary Materials

Effectiveness and safety of direct oral anticoagulants for secondary prevention in patients with non-valvular atrial fibrillation

Tables

Table S1. Definition of covariates and study outcomes

Table S2. Characteristics of study population after IPTW adjustment

Table S3. Subgroup analysis of clinical outcomes according to antithrombotic therapy

Table S4. Crude and multivariate-adjusted risk of clinical outcomes according to antithrombotic therapy

Table S5. Crude and multivariate-adjusted risk of clinical outcomes according to antithrombotic therapy (Warfarin vs 4 DOACs)

Table S6. Characteristics of the study population with warfarin and dosage of DOAC

Table S7. Comparison of clinical outcomes between patients with warfarin and regular dose DOAC

Figures

Figure S1. Study flow

Figure S2. Distribution of propensity scores before and after IPTW

Figure S3. Kaplan-Meier curve of clinical outcomes according to antithrombotic therapy (Warfarin vs. pooled DOACs)

Figure S4. Kaplan-Meier curve of clinical outcomes according to antithrombotic therapy (Warfarin vs. 4 DOACs)

Figure S5. Subgroup analysis of clinical outcomes according to antithrombotic therapy

Figure S6. Kaplan-Meier curve of clinical outcomes among patients with warfarin and regular dose DOAC

Figure S7. Comparison of clinical outcomes between patients with warfarin and 4 regular dose DOACs

Figure S8. Kaplan-Meier curve of clinical outcomes among patients with warfarin and four different regular dose DOAC

Figure S9. Sensitivity analysis comparing clinical outcomes according to antithrombotic therapy during a 1-year follow-up

Table S1. Definition of covariates and study outcomes

Diagnosis	ICD-10-CM code and definition	Diagnostic definition
**Inclusion/exclusion criteria**		
**Atrial fibrillation**	I48.0-48.4, I48.9	Admission or outpatient department≥1
**Valvular atrial fibrillation**	I05.0, I05.2, I05.9, Z95.2-Z95.4	
**Pulmonary embolism**	I26	Admission≥1
**Deep vein thrombosis**	I80.2	Admission≥1
**Received joint replacement operation**	N0711, N1711, N1721, N2070, N3710, N3721, N3717, N3720, N2072, N2077, N3722, N3727	Admission≥1
**End stage renal disease**	N18.5, Z49	Dialysis≥2
**Comorbidities**		
**Hypertension**	I10-I13, I15; and minimum 1 prescription of anti-hypertensive drug (thiazide, loop diuretics, aldosterone antagonist, alpha-/beta-blocker, calcium-channel blocker, angiotensin-converting enzyme inhibitor, angiotensin II receptor blocker).	Admission≥1 or outpatient department≥2
**Diabetes mellitus**	E11-E14; and minimum 1 prescription of anti-diabetic drugs (sulfonylureas, metformin, meglitinides, thiazolidinediones, dipeptidyl peptidase-4 inhibitors, α-glucosidase inhibitors, and insulin).	Admission≥1 or outpatient department≥2
**Dyslipidemia**	E78	Admission or outpatient department≥1
**Heart failure**	I50	Admission or outpatient department≥1
**Vascular disease**		
**Prior MI**	I21, I22	Admission or outpatient department≥1
**PAD**	I70, I73	Admission or outpatient department≥2
**Renal disease**	I13.1, N03, N05, N10-N19, Z49, Z94.0, Z99.2	Admission or outpatient department≥1
**Liver disease**	K70, K72-K76, K71.3-K71.7	Admission or outpatient department≥1
**COPD**	J41-44	Admission≥1
**Cancer**	C00-97 and RID code (V193)	Admission or outpatient department≥1
**Scores**		
**CHA_2DS_2-VASc score**	Heart failure (1 point), hypertension (1 point), age ≥75 years (2 points), diabetes (1 point), previous stroke/systemic embolism/transient ischemic attack (2 points), vascular disease (prior MI or PAD, 1 point) and female sex (1 point)	
**HAS-BLED score***	Hypertension (1 point), liver disease (1 point), renal disease (1 point), stroke history (1 point), bleeding history (1 point), age>65 years (1 point) and drug (concomitant use of NSAID or antiplatelet agent, 1 point)	

Clinical outcome		
**Recurrent stroke**	I63, I64	Primary diagnosis, admission≥1 (≥3 days) and brain imaging (CT or MRI) ≥1
**Intracranial hemorrhage**	I60-62	Primary diagnosis, admission≥1 (≥3 days) and brain imaging (CT or MRI) ≥1
**Gastrointestinal bleeding**		
**Major bleeding**	Intracranial hemorrhage + gastrointestinal bleeding	
**Composite outcome**	Recurrent stroke + major bleeding	
**Fatal recurrent stroke**	In-hospital death from "Recurrent stroke"	
**Fatal major bleeding**	In-hospital death from "Major bleeding"	
**Fatal composite outcome**	In-hospital death from "Recurrent stroke + major bleeding"	
**All-cause death**	In-hospital death from any causes	

Abbreviation: COPD, chronic obstructive pulmonary disease; MI, myocardial infarction; NSAID, non-steroidal anti-inflammatory drug; PAD, peripheral artery disease; RID, Rare Intractable Disease program.

*Labile international normalized ratio (INR) and alcohol use could not be evaluated from claims and were excluded from scoring in this study.

Table S2. Characteristics of study population after IPTW adjustment

	Warfarin	DOAC	ASD	Rivaroxaban	Dabigatran	Apixaban	Edoxaban	ASD
**Male**	15100 (52.2)	17042 (52.1)	0.003	6399 (52.1)	3284 (52.1)	4583 (52.0)	2776 (52.1)	0.002
**Age**			0.001					0.003
Mean±SD	74.2±9.1	74.2±9.5		74.3±9.3	74.3±9.6	74.1±9.8	74.4±9.4	
Median (IQR)	75 (69-81)	75 (69-81)		75 (69-81)	75 (69-81)	75 (68-81)	75 (69-81)	
<65 years	3863 (13.4)	4899 (15.0)		1824 (14.9)	929 (14.7)	1361 (15.5)	785 (14.7)	
65-74 years	9784 (33.8)	10096 (30.9)		3795 (30.9)	1960 (31.1)	2687 (30.5)	1654 (31.0)	
75≤ years	15272 (52.8)	17729 (54.2)		6663 (54.3)	3414 (54.2)	4760 (54.0)	2892 (54.2)	
**CHA_2DS_2-VASc score**			0.002					0.005
Mean±SD	5.9±1.5	5.9±1.4		5.9±1.4	5.9±1.5	5.8±1.4	5.9±1.5	
Median (IQR)	6 (5-7)	6 (5-7)		6 (5-7)	6 (5-7)	6 (5-7)	6 (5-7)	
2	274 (1.0)	346 (1.1)		105 (0.9)	76 (1.2)	111 (1.3)	55 (1.0)	
3≤	28645 (99.0)	32378 (98.9)		12178 (99.1)	6226 (98.8)	8697 (98.7)	5277 (99.0)	
**HAS-BLED score**			0.002					0.003
Mean±SD	4.23±1.07	4.23±1.1		4.2±1.1	4.2±1.1	4.2±1.1	4.3±1.1	
Median (IQR)	4 (4-5)	4 (4-5)		4 (4-5)	4 (4-5)	4 (4-5)	4 (4-5)	
3≤	27570 (95.3)	31036 (94.8)		11626 (94.7)	5994 (95.1)	8356 (94.9)	5061 (94.9)	
**CCI**			0.001					0.001
Mean±SD	5.01±2.49	5.0±2.6		5.0±2.6	5.0±2.6	5.0±2.5	5.1±2.6	
Median (IQR)	5 (3-7)	5 (3-7)		5 (3-7)	5 (3-7)	5 (3-7)	5 (3-7)	

3≤	24261 (83.9)	27270 (83.3)		10217 (83.2)	5274 (83.7)	7353 (83.5)	4427 (83.0)	
**Hypertension**	25961 (89.8)	29429 (89.9)	0.005	11072 (90.1)	5669 (89.0)	7896 (89.6)	4793 (89.9)	0.012
**Diabetes mellitus**	9577 (33.1)	10867 (33.2)	0.002	4048 (33.0)	2108 (33.5)	2921 (33.2)	1790 (33.6)	0.003
**Dyslipidemia**	19192 (66.4)	21759 (66.5)	0.003	8172 (66.5)	4154 (65.9)	5865 (66.6)	3567 (66.9)	0.004
**Congestive heart failure**	12622 (43.7)	14292 (43.7)	0.001	5404 (44.0)	2726 (43.3)	3840 (43.6)	2322 (43.6)	0.007
**Myocardial infarction**	1703 (5.9)	1928 (5.9)	<0.001	725 (5.9)	374 (5.9)	519 (5.9)	310 (5.8)	0.001
**Peripheral arterial disease**	7905 (27.3)	8919 (27.3)	0.002	3341 (27.2)	1716 (27.2)	2385 (27.1)	1477 (27.7)	0.003
**COPD**	3819 (13.2)	4317 (13.2)	<0.001	1636 (13.3)	832 (13.2)	1134 (12.9)	716 (13.4)	0.003
**Cancer**	2176 (7.5)	2419 (7.4)	0.005	907 (7.4)	463 (7.4)	663 (7.5)	386 (7.2)	0.005
**Previous Intracranial hemorrhage**	1617 (5.6)	1859 (5.7)	0.004	695 (5.7)	352 (5.6)	499 (5.7)	312 (5.9)	0.003
**Previous gastrointestinal bleeding**	6061 (21.0)	6780 (20.7)	0.006	2546 (20.7)	1315 (20.9)	1826 (20.7)	1094 (20.5)	0.006
**Antiplatelets**			0.002					0.001
No antiplatelets	16133 (55.8)	18216 (55.7)		6849 (55.8)	3496 (55.5)	4971 (56.4)	2901 (54.4)	
Aspirin only	6160 (21.3)	7003 (21.4)		2594 (21.1)	1360 (21.6)	1860 (21.1)	1189 (22.3)	
Clopidogrel only	2802 (9.7)	3195 (9.8)		1202 (9.8)	608 (9.6)	853 (9.7)	532 (10.0)	
Dual antiplatelets	3823 (13.2)	4309 (13.2)		1637 (13.3)	838 (13.3)	1124 (12.8)	710 (13.3)	
**NSAID**	13232 (45.8)	14923 (45.6)	0.003	5607 (45.7)	2886 (45.8)	4016 (45.6)	2413 (45.3)	0.002
**Proton pump inhibitors**	10022 (34.7)	11275 (34.5)	0.004	4273 (34.8)	2157 (34.2)	3017 (34.3)	1828 (34.3)	0.003

The numbers are presented as mean ± standard deviation, median (IQR), or numbers (percentage) otherwise mentioned.
Abbreviation: ASD, absolute standardized difference; CCI, charlson comorbidity index; COPD, chronic obstructive pulmonary disease; DOAC, direct oral anticoagulant; IPTW, inverse probability treatment weighting; IQR, interquartile range; NSAID, non-steroidal anti-inflammatory drug; SD, standard deviation.

Table S3. Subgroup analysis of clinical outcomes according to antithrombotic therapy

Subgroups		Total number	Recurrent stroke	Major bleeding	Composite outcome	All-cause death	Fatal recurrent stroke	Fatal major bleeding	Fatal composite outcome
**Sex**									
**Male**	Warfarin	15338	1195 (4.4)	689 (2.4)	1793 (6.7)	1556 (5.4)	201 (0.7)	92 (0.3)	290 (1.0)
	DOAC	16703	533 (3.6)	309 (2.1)	816 (5.6)	1071 (7.2)	124 (0.8)	26 (0.2)	150 (1.0)
**Female**	Warfarin	13501	1227 (5.6)	606 (2.7)	1747 (8.2)	1493 (6.5)	273 (1.2)	105 (0.5)	375 (1.6)
	DOAC	16026	552 (4.3)	301 (2.3)	833 (6.5)	1059 (8.1)	171 (1.3)	32 (0.2)	201 (1.5)
**Age (years)**									
**≥75**	Warfarin	13827	1141 (6.2)	614 (3.2)	1678 (9.4)	2001 (10.4)	313 (1.6)	120 (0.6)	431 (2.2)
	DOAC	18980	622 (4.4)	403 (2.8)	994 (7.1)	1706 (12.0)	227 (1.6)	40 (0.3)	266 (1.8)
**<75**	Warfarin	15012	1281 (4.1)	681 (2.1)	1862 (6.2)	1048 (3.2)	161 (0.5)	77 (0.2)	234 (0.7)
	DOAC	13749	463 (3.4)	207 (1.5)	655 (4.9)	424 (3.1)	68 (0.5)	18 (0.1)	85 (0.6)
**CHA_2DS_2-VASc**									
**≥3**	Warfarin	28506	2397 (4.9)	1288 (2.6)	3509 (7.4)	3042 (5.9)	474 (0.9)	197 (0.4)	665 (1.3)
	DOAC	32436	1082 (4.0)	609 (2.2)	1645 (6.1)	2125 (7.7)	292 (1.0)	58 (0.2)	348 (1.2)
**<3**	Warfarin	333	25 (3.4)	7 (0.9)	31 (4.2)	7 (0.9)	0 (0.0)	0 (0.0)	0 (0.0)
	DOAC	293	3 (1.0)	1 (0.3)	4 (1.3)	5 (1.6)	3 (1.0)	0 (0.0)	3 (1.0)
**HAS-BLED**									
**≥3**	Warfarin	27534	2325 (4.9)	1256 (2.6)	3410 (7.4)	2971 (5.9)	454 (0.9)	196 (0.4)	644 (1.3)
	DOAC	30997	1043 (4.0)	597 (2.2)	1594 (6.1)	2064 (7.7)	285 (1.1)	55 (0.2)	338 (1.3)
**<3**	Warfarin	1305	97 (5.1)	39 (1.9)	130 (6.9)	78 (3.8)	20 (1.0)	1 (0.0)	21 (1.0)
	DOAC	1732	42 (3.3)	13 (1.0)	55 (4.3)	66 (5.1)	10 (0.8)	3 (0.2)	13 (1.0)
**Diabetes**									
**Yes**	Warfarin	9869	846 (5.4)	469 (2.9)	1253 (8.2)	1244 (7.5)	172 (1.0)	74 (0.4)	244 (1.5)
	DOAC	10467	408 (4.9)	209 (2.5)	596 (7.2)	805 (9.4)	111 (1.3)	12 (0.1)	123 (1.4)
**No**	Warfarin	18970	1576 (4.7)	826 (2.4)	2287 (7.0)	1805 (5.1)	302 (0.8)	123 (0.3)	421 (1.2)
	DOAC	22262	677 (3.5)	401 (2.1)	1053 (5.5)	1325 (6.8)	184 (0.9)	46 (0.2)	228 (1.2)

**Hypertension**									
**Yes**	Warfarin	26072	2235 (5.0)	1208 (2.6)	3276 (7.5)	2794 (5.9)	433 (0.9)	184 (0.4)	613 (1.3)
	DOAC	29265	1014 (4.1)	573 (2.3)	1542 (6.3)	1923 (7.6)	273 (1.1)	50 (0.2)	321 (1.3)
**No**	Warfarin	2767	187 (4.0)	87 (1.8)	264 (5.7)	255 (5.1)	41 (0.8)	13 (0.3)	52 (1.0)
	DOAC	3464	71 (2.6)	37 (1.3)	107 (3.9)	207 (7.4)	22 (0.8)	8 (0.3)	30 (1.1)
**Heart failure**									
**Yes**	Warfarin	11972	987 (5.3)	532 (2.8)	1444 (8.0)	1474 (7.5)	197 (1.0)	76 (0.4)	271 (1.4)
	DOAC	14780	485 (4.1)	297 (2.5)	759 (6.5)	1094 (9.1)	114 (0.9)	33 (0.3)	146 (1.2)
**No**	Warfarin	16867	1435 (4.7)	763 (2.4)	2096 (7.0)	1575 (4.8)	277 (0.8)	121 (0.4)	394 (1.2)
	DOAC	17949	600 (3.8)	313 (2.0)	890 (5.7)	1036 (6.5)	181 (1.1)	25 (0.2)	205 (1.3)
**CKD**									
**Yes**	Warfarin	5079	356 (5.6)	255 (3.9)	583 (9.4)	854 (12.8)	95 (1.4)	40 (0.6)	132 (2.0)
	DOAC	5438	193 (5.2)	152 (4.0)	334 (9.1)	631 (16.7)	53 (1.4)	15 (0.4)	68 (1.8)
**No**	Warfarin	23760	2066 (4.8)	1040 (2.3)	2957 (7.1)	2195 (4.8)	379 (0.8)	157 (0.3)	533 (1.2)
	DOAC	27291	892 (3.7)	458 (1.9)	1315 (5.6)	1499 (6.2)	242 (1.0)	43 (0.2)	283 (1.2)
**CCI**									
**≥3**	Warfarin	24466	2173 (5.5)	1144 (2.8)	3161 (8.1)	2780 (6.5)	424 (1.0)	172 (0.4)	591 (1.4)
	DOAC	26808	997 (4.5)	555 (2.5)	1508 (7.0)	1979 (8.9)	267 (1.2)	49 (0.2)	314 (1.4)
**<3**	Warfarin	4373	249 (2.6)	151 (1.6)	379 (4.1)	269 (2.8)	50 (0.5)	25 (0.3)	74 (0.8)
	DOAC	5921	88 (1.6)	55 (1.0)	141 (2.5)	151 (2.6)	28 (0.5)	9 (0.2)	37 (0.6)

Number of events (crude incidence rates, per 100 person-years)

Abbreviation: CCI, charlson comorbidity index; CKD, chronic kidney disease; DOAC, direct oral anticoagulant.

Table S4. Crude and multivariate-adjusted risk of clinical outcomes according to antithrombotic therapy

Outcome	Warfarin Event	Warfarin IR	DOAC Event	DOAC IR	Crude HR (95% CI)	p-value	Multivariate adjusted HR (95% CI)	p-value
**Recurrent stroke**	2422	4.9	1085	3.9	0.61 (0.57-0.66)	<0.001	0.66 (0.61-0.71)	<0.001
**Major bleeding**	1295	2.5	610	2.2	0.73 (0.66-0.81)	<0.001	0.72 (0.66-0.81)	<0.001
**Composite outcome (Recurrent stroke + Major bleeding)**	3540	7.4	1649	6.0	0.65 (0.61-0.69)	<0.001	0.68 (0.64-0.72)	<0.001
**All-cause death**	3049	5.9	2130	7.6	0.95 (0.89-1.00)	0.069	0.84 (0.79-0.89)	<0.001
**Fatal recurrent stroke**	474	0.9	295	1.0	0.72 (0.62-0.84)	<0.001	0.65 (0.55-0.75)	<0.001
**Fatal major bleeding**	197	0.4	58	0.2	0.49 (0.36-0.67)	<0.001	0.47 (0.35-0.65)	<0.001
**Fatal composite outcome**	665	1.3	351	1.2	0.67 (0.58-0.77)	<0.001	0.61 (0.53-0.69)	<0.001

Abbreviation: CI, confidence interval; HR, hazard ratio; IR, incidence rate; DOAC, direct oral anticoagulant.

Table S5. Crude and multivariate-adjusted risk of clinical outcomes according to antithrombotic therapy (Warfarin vs 4 DOACs)

Group	Recurrent stroke	IR	Crude HR (95% CI)	Multivariate adjusted HR (95% CI)
**Warfarin**	2422	4.9	1(Ref.)	1(Ref.)
**Rivaroxaban**	388	3.6	0.57 (0.51-0.63)	0.62 (0.55-0.69)
**Dabigatran**	260	4.1	0.68 (0.60-0.78)	0.76 (0.67-0.86)
**Apixaban**	322	4.5	0.69 (0.61-0.78)	0.71 (0.63-0.80)
**Edoxaban**	115	3.5	0.47 (0.39-0.57)	0.49 (0.41-0.60)
**Group**	**Major bleeding**	**IR**	**Crude HR (95% CI)**	**Multivariate adjusted HR (95% CI)**
**Warfarin**	1295	2.5	1(Ref.)	1(Ref.)
**Rivaroxaban**	264	2.4	0.81 (0.71-0.93)	0.82 (0.71-0.94)
**Dabigatran**	141	2.2	0.77 (0.64-0.91)	0.82 (0.68-0.97)
**Apixaban**	149	2.1	0.68 (0.57-0.81)	0.64 (0.54-0.76)
**Edoxaban**	56	1.7	0.51 (0.39-0.67)	0.50 (0.38-0.65)
**Group**	**Composite outcome**	**IR**	**Crude HR (95% CI)**	**Multivariate adjusted HR (95% CI)**
**Warfarin**	3540	7.4	1(Ref.)	1(Ref.)
**Rivaroxaban**	635	5.9	0.65 (0.60-0.71)	0.69 (0.63-0.75)
**Dabigatran**	385	6.2	0.70 (0.63-0.78)	0.76 (0.69-0.85)
**Apixaban**	461	6.5	0.69 (0.63-0.76)	0.69 (0.62-0.76)
**Edoxaban**	168	5.1	0.48 (0.41-0.57)	0.49 (0.42-0.58)
**Group**	**All-cause death**	**IR**	**Crude HR (95% CI)**	**Multivariate adjusted HR (95% CI)**
**Warfarin**	3049	5.9	1(Ref.)	1(Ref.)
**Rivaroxaban**	889	8.0	1.04 (0.96-1.12)	0.92 (0.85-0.99)
**Dabigatran**	344	5.4	0.72 (0.65-0.81)	0.75 (0.67-0.84)
**Apixaban**	647	8.9	1.09 (0.99-1.19)	0.86 (0.79-0.94)
**Edoxaban**	250	7.5	0.78 (0.69-0.89)	0.69 (0.60-0.78)
**Group**	**Fatal recurrent stroke**	**IR**	**Crude HR (95% CI)**	**Multivariate adjusted HR (95% CI)**
**Warfarin**	474	0.9	1(Ref.)	1(Ref.)
**Rivaroxaban**	121	1.1	0.78 (0.63-0.95)	0.71 (0.58-0.87)
**Dabigatran**	61	1.0	0.73 (0.55-0.95)	0.73 (0.55-0.95)
**Apixaban**	85	1.2	0.78 (0.62-0.98)	0.64 (0.50-0.81)
**Edoxaban**	28	0.8	0.46 (0.31-0.67)	0.40 (0.27-0.58)
**Group**	**Fatal major bleeding**	**IR**	**Crude HR (95% CI)**	**Multivariate adjusted HR (95% CI)**
**Warfarin**	197	0.4	1(Ref.)	1(Ref.)
**Rivaroxaban**	30	0.3	0.65 (0.44-0.96)	0.63 (0.42-0.93)
**Dabigatran**	5	0.1	0.19 (0.08-0.47)	0.20 (0.08-0.49)
**Apixaban**	15	0.2	0.49 (0.29-0.83)	0.44 (0.26-0.76)
**Edoxaban**	8	0.2399	0.53 (0.26-1.09)	0.50 (0.24-1.02)
**Group**	**Fatal composite outcome**	**IR**	**Crude HR (95% CI)**	**Multivariate adjusted HR (95% CI)**
**Warfarin**	665	1.3	1(Ref.)	1(Ref.)
**Rivaroxaban**	149	1.3	0.74 (0.62-0.89)	0.69 (0.57-0.82)
**Dabigatran**	66	1.0	0.60 (0.47-0.78)	0.61 (0.47-0.79)
**Apixaban**	100	1.4	0.72 (0.58-0.89)	0.60 (0.48-0.75)
**Edoxaban**	36	1.1	0.47 (0.34-0.67)	0.41 (0.30-0.58)

Abbreviation: CI, confidence interval; HR, hazard ratio; IR, incidence rate; DOAC, direct oral anticoagulant.

Table S6. Characteristics of the study population with warfarin and dosage of DOAC

	(A) Warfarin (n=28839)	(B) Regular dose DOAC (n=12073)	(C) Reduced dose DOAC (n=20656)	A vs. B p value	A vs. C p value	B vs. C p value
**Age**						
Mean±SD	73.1±9.4	71.7±9.4	77.3±8.3	<0.001	<0.001	<0.001
Median (IQR)	74 (68-79)	73 (66-78)	78 (73-83)	<0.001	<0.001	<0.001
<65 years	4757 (16.5)	2509 (20.8)	1465 (7.1)	<0.001	<0.001	<0.001
65-74 years	10255 (35.6)	4612 (38.2)	5163 (25.0)			
75≤ years	13827 (48.0)	4952 (41.0)	14028 (67.9)			
**Male**	15338 (53.2)	7269 (60.2)	11222 (54.3)	<0.001	<0.001	<0.001
**CHA_2DS_2-VASc score**						
Mean±SD	5.8±1.5	5.5±1.5	6.2±8.2	<0.001	<0.001	<0.001
2	333 (1.2)	221 (1.8)	72 (0.4)	<0.001	<0.001	<0.001
3≤	28506 (98.8)	11852 (98.1)	20584 (99.6)			
**HAS-BLED score**						
Mean±SD	4.2±1.1	4.0±1.1	4.3±1.1	<0.001	<0.001	<0.001
3≤	27534 (95.5)	11103 (92.0)	19894 (96.3)	<0.001	<0.001	<0.001
**CCI**						
Mean±SD	5.2±2.5	4.7±2.5	5.0±2.5	<0.001	<0.001	<0.001
3≤	24466 (84.8)	9566 (79.2)	17242 (83.5)	<0.001	<0.001	<0.001
**Hypertension**	26072 (90.4)	10633 (88.1)	18632 (90.2)	<0.001	0.449	<0.001

**Diabetes mellitus**	9869 (34.2)	3844 (31.8)	6623 (32.1)	<0.001	<0.001	0.676
**Dyslipidemia**	18774 (65.1)	8412 (69.7)	13487 (65.3)	<0.001	0.655	<0.001
**Congestive heart failure**	11972 (41.5)	5028 (41.7)	9752 (47.2)	0.803	<0.001	<0.001
**Myocardial infarction**	1836 (6.4)	546 (4.5)	1283 (6.2)	<0.001	0.484	<0.001
**Peripheral arterial disease**	7668 (26.6)	3133 (26.0)	5942 (28.8)	0.181	<0.001	<0.001
**COPD**	4080 (14.2)	1293 (10.7)	2701 (13.1)	<0.001	<0.001	<0.001
**Cancer**	2000 (6.9)	912 (7.6)	1629 (7.9)	0.026	<0.001	0.278
**Previous Intracranial hemorrhage**	1530 (5.3)	673 (5.6)	1286 (6.23)	0.271	<0.001	0.017
**Previous gastrointestinal bleeding**	5039 (17.5)	2684 (22.3)	4994 (24.2)	<0.001	<0.001	<0.001
**Antiplatelets**						
No antiplatelets	12731 (44.2)	8199 (67.9)	13255 (64.2)	<0.001	<0.001	<0.001
Aspirin only	7990 (27.7)	1793 (14.9)	3390 (16.4)			
Clopidogrel only	2816 (9.8)	1029 (8.5)	2180 (10.6)			
Dual antiplatelets	5302 (18.4)	1052 (8.7)	1831 (8.9)			
**NSAID**	13940 (48.3)	5440 (45.1)	8881 (43.0)	<0.001	<0.001	<0.001
**Proton pump inhibitors**	9090 (31.5)	4374 (36.2)	7699 (37.3)	<0.001	<0.001	0.059

The numbers are presented as mean ± standard deviation, median (IQR), or numbers (percentage) otherwise mentioned.
Abbreviation: ASD, absolute standardized difference; CCI, charlson comorbidity index; COPD, chronic obstructive pulmonary disease; DOAC, direct oral anticoagulant; IPTW, inverse probability treatment weighting; IQR, interquartile range; NSAID, non-steroidal anti-inflammatory drug; SD, standard deviation.

Table S7. Comparison of clinical outcomes between patients with warfarin and regular dose DOAC

	Warfarin	Regular dose DOAC		
**Outcome**	**Event (IR)**	**Event (IR)**	***HR (95% CI)**	**p-value**
**Recurrent stroke**	2362 (4.8)	477 (4.4)	0.70 (0.63-0.77)	<0.001
**Major bleeding**	1269 (2.5)	214 (1.9)	0.66 (0.57-0.76)	<0.001
**Composite outcome (Recurrent stroke + Major bleeding)**	3456 (7.2)	675 (6.2)	0.69 (0.63-0.75)	<0.001
**Fatal recurrent stroke**	463 (0.9)	99 (0.9)	0.64 (0.51-0.80)	<0.001
**Fatal major bleeding**	187 (0.4)	15 (0.1)	0.34 (0.19-0.56)	<0.001
**Fatal composite outcome**	645 (1.2)	114 (1.0)	0.58 (0.47-0.71)	<0.001
**All-cause death**	2958 (5.7)	651 (5.8)	0.76 (0.70-0.83)	<0.001

* IPTW adjustment

Abbreviation: CI, confidence interval; DOAC, direct oral anticoagulant; HR, hazard ratio; IR, incidence rate.

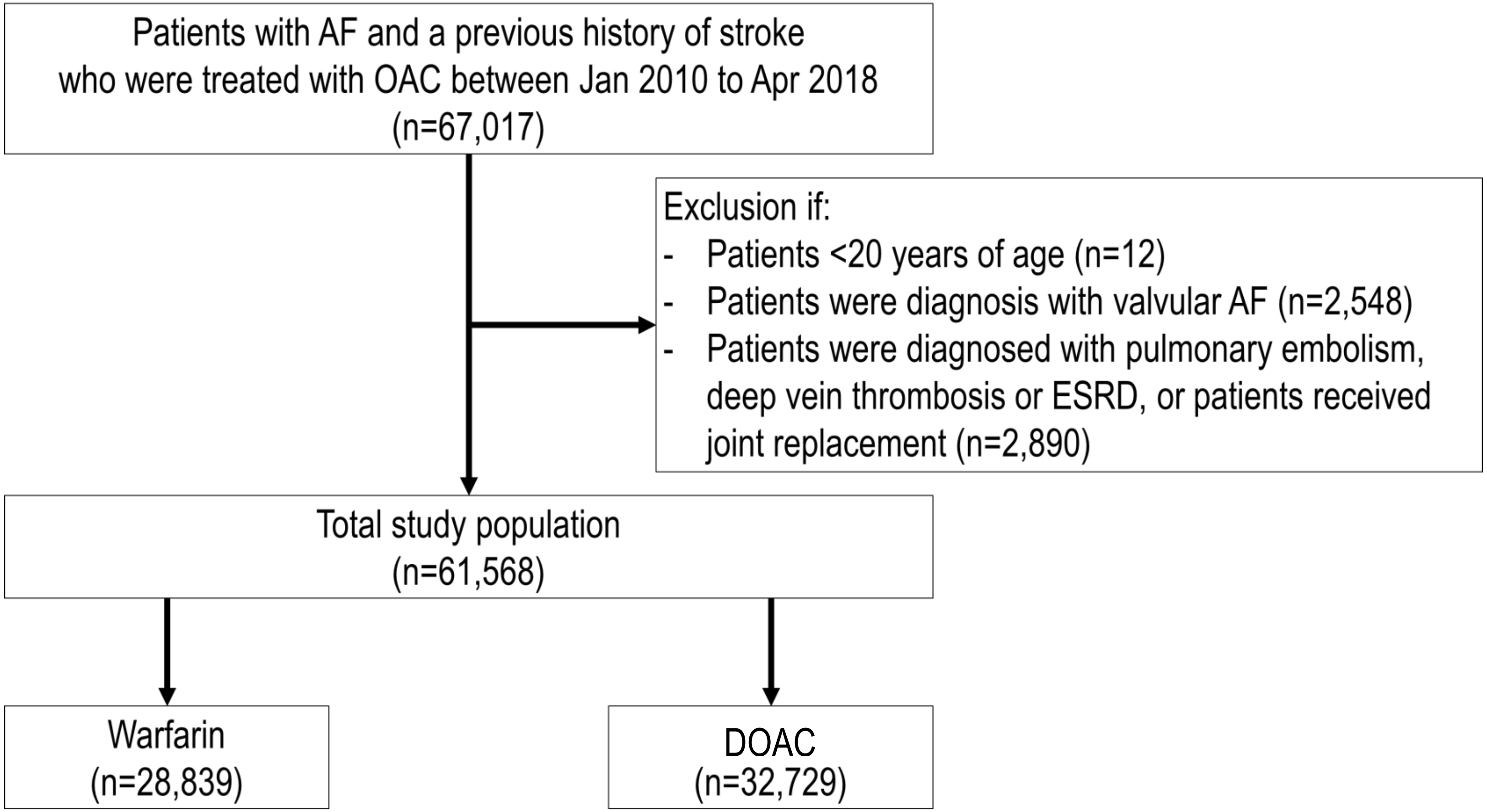

Figure S1. Study flow

AF = atrial fibrillation, DOAC = direct oral anticoagulant, ESRD = end stage renal disease, OAC = oral anticoagulant.

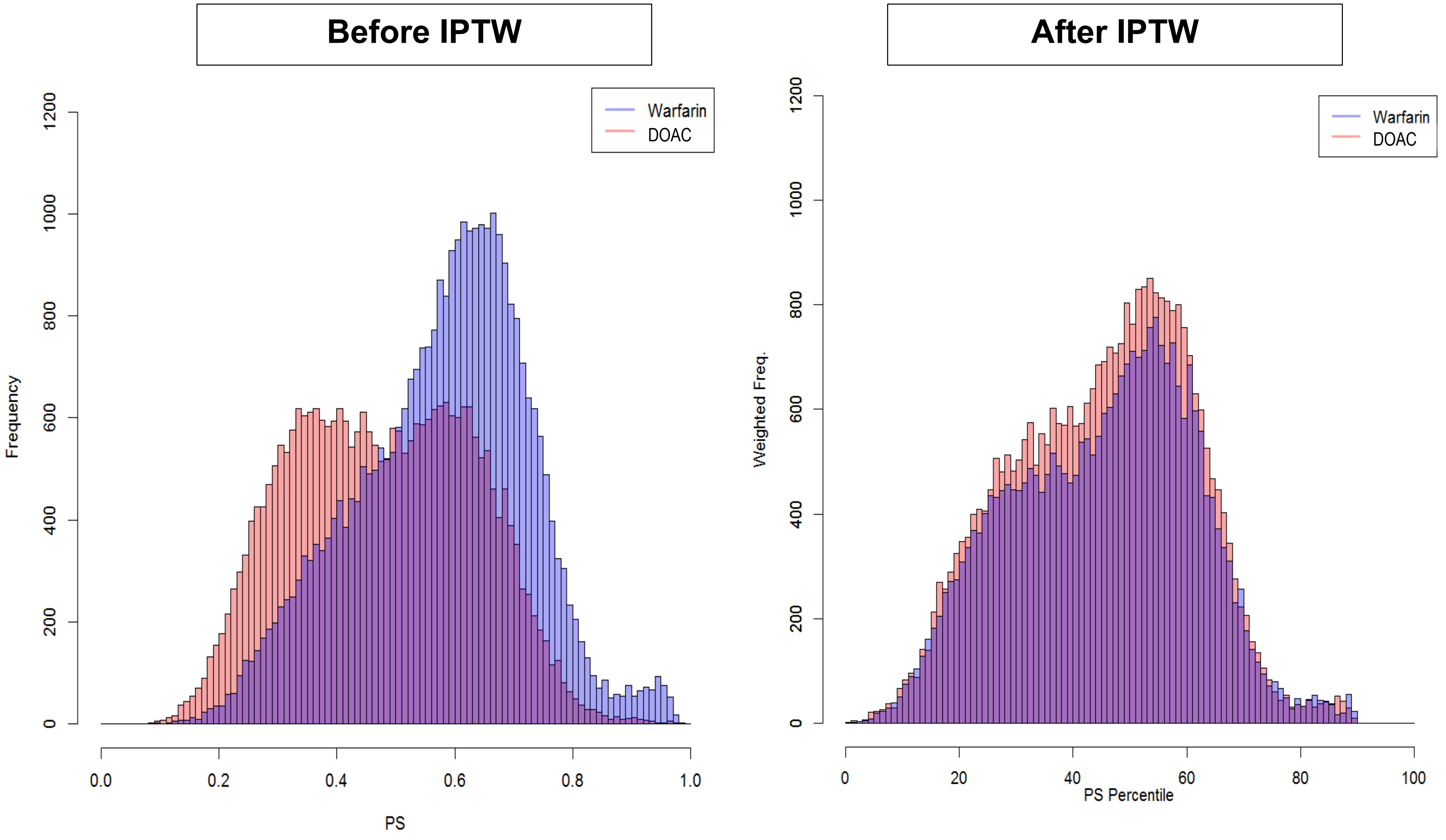

Figure S2. Distribution of propensity scores before and after IPTW

DOAC = direct oral anticoagulant, IPTW = inverse probability of treatment weights, PS = propensity score

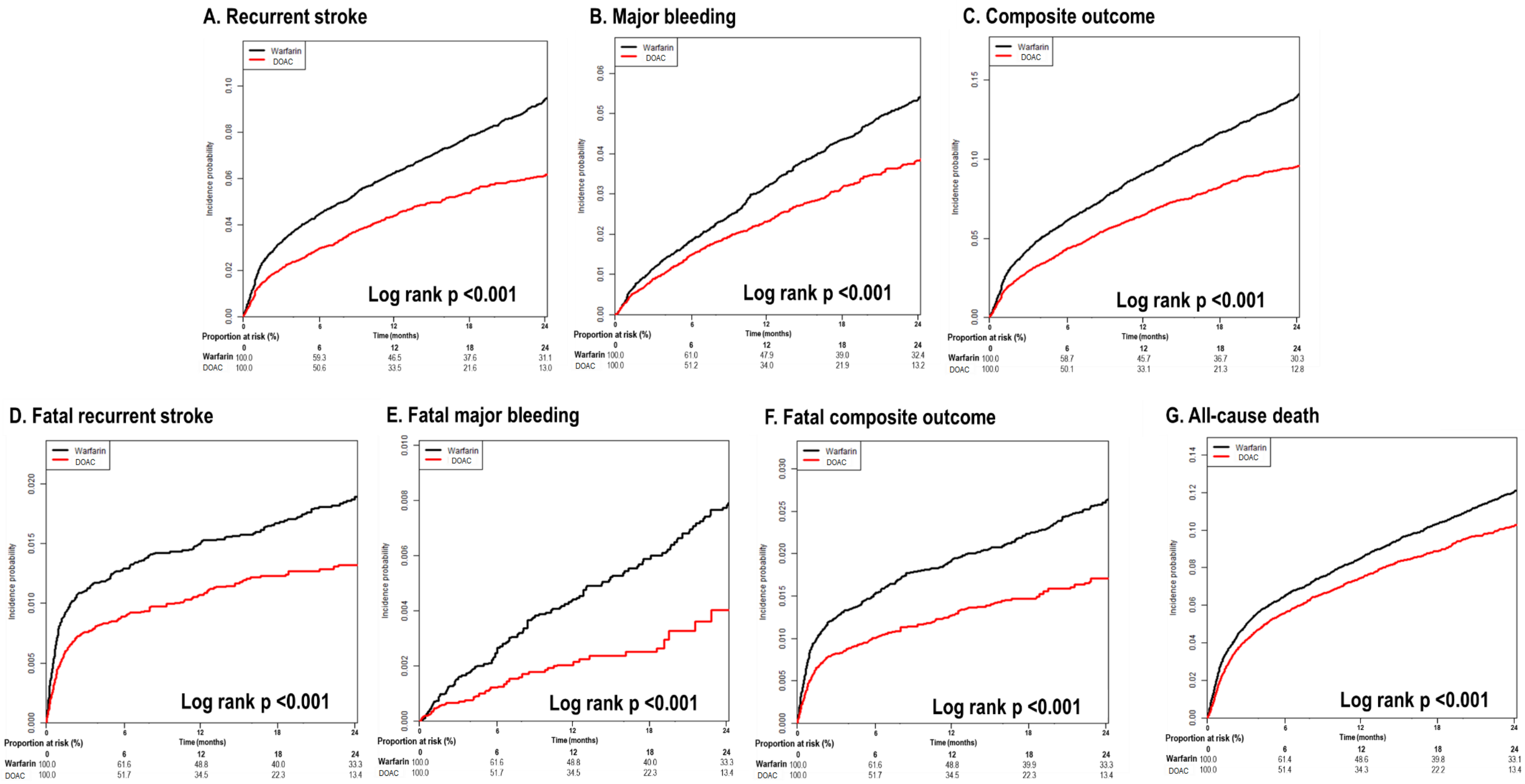

Figure S3. Kaplan-Meier curve of clinical outcomes according to antithrombotic therapy (Warfarin vs. pooled DOACs)

DOAC = direct oral anticoagulant

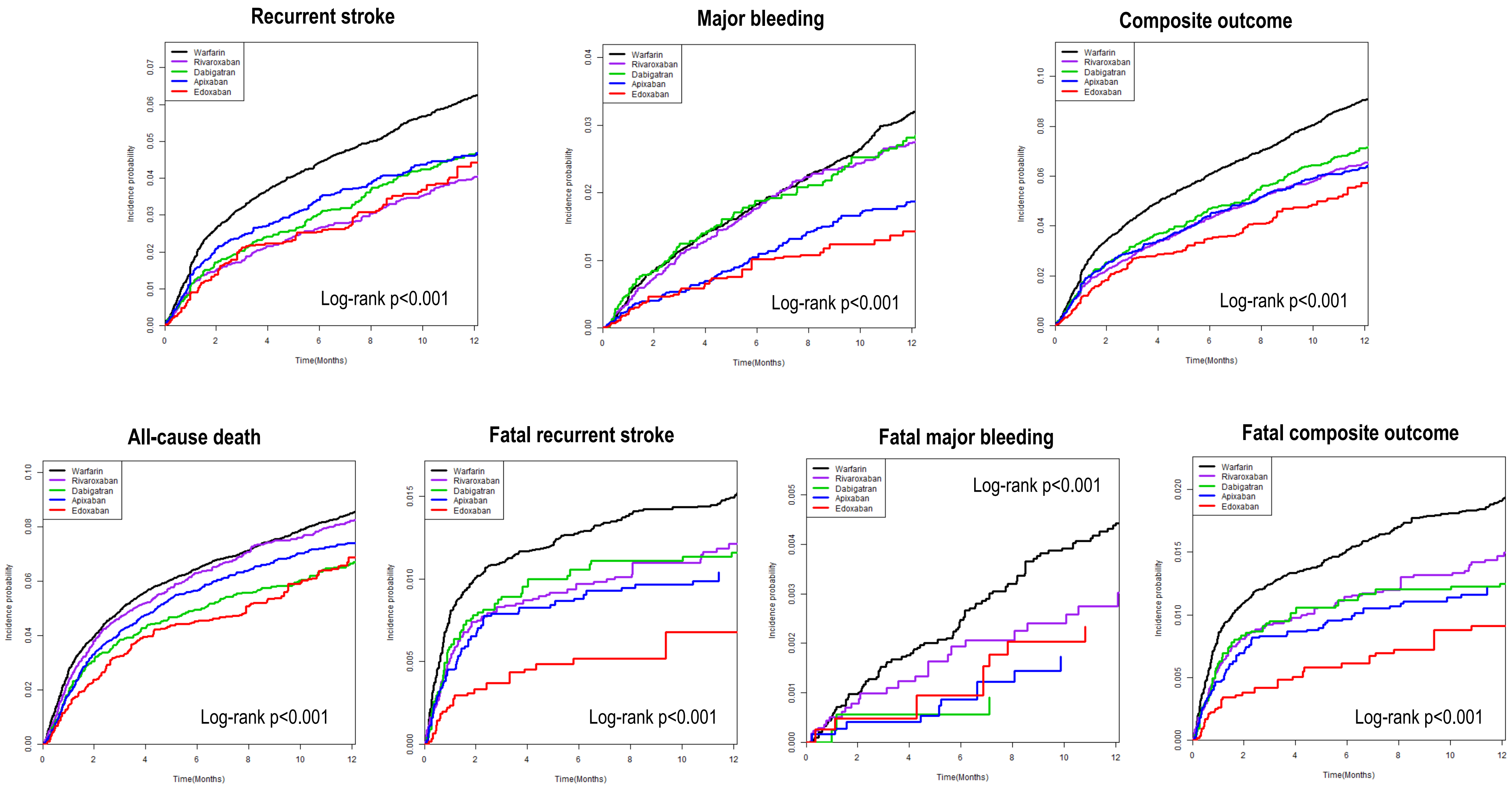

Figure S4. Kaplan-Meier curve of clinical outcomes according to antithrombotic therapy (Warfarin vs. 4 DOACs)

DOAC = direct oral anticoagulant

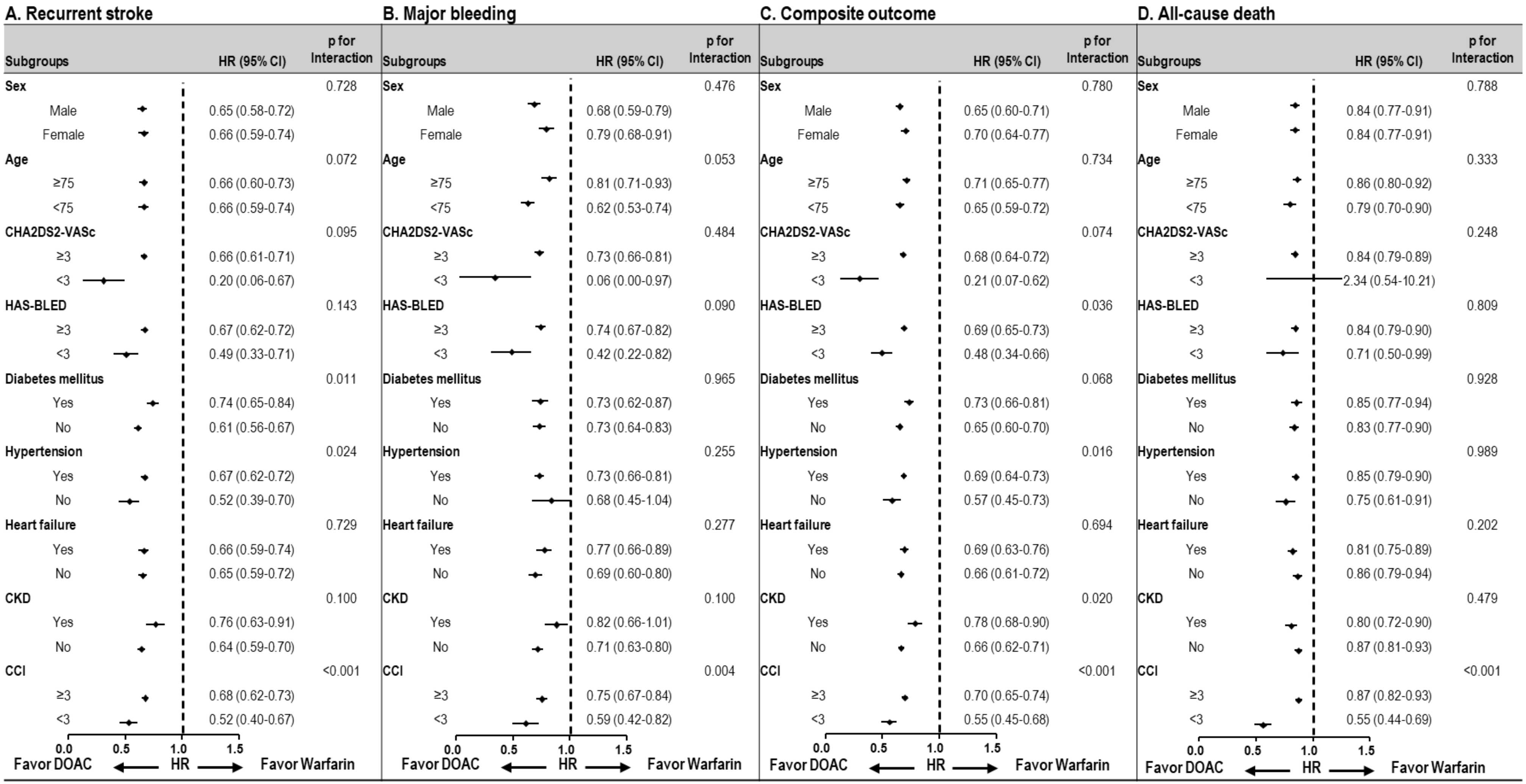

Figure S5. Subgroup analysis of clinical outcomes according to antithrombotic therapy

CCI = charlson comorbidity index, CI = confidence interval, CKD = chronic kidney disease, DOAC = direct oral anticoagulant, HR = hazard ratio

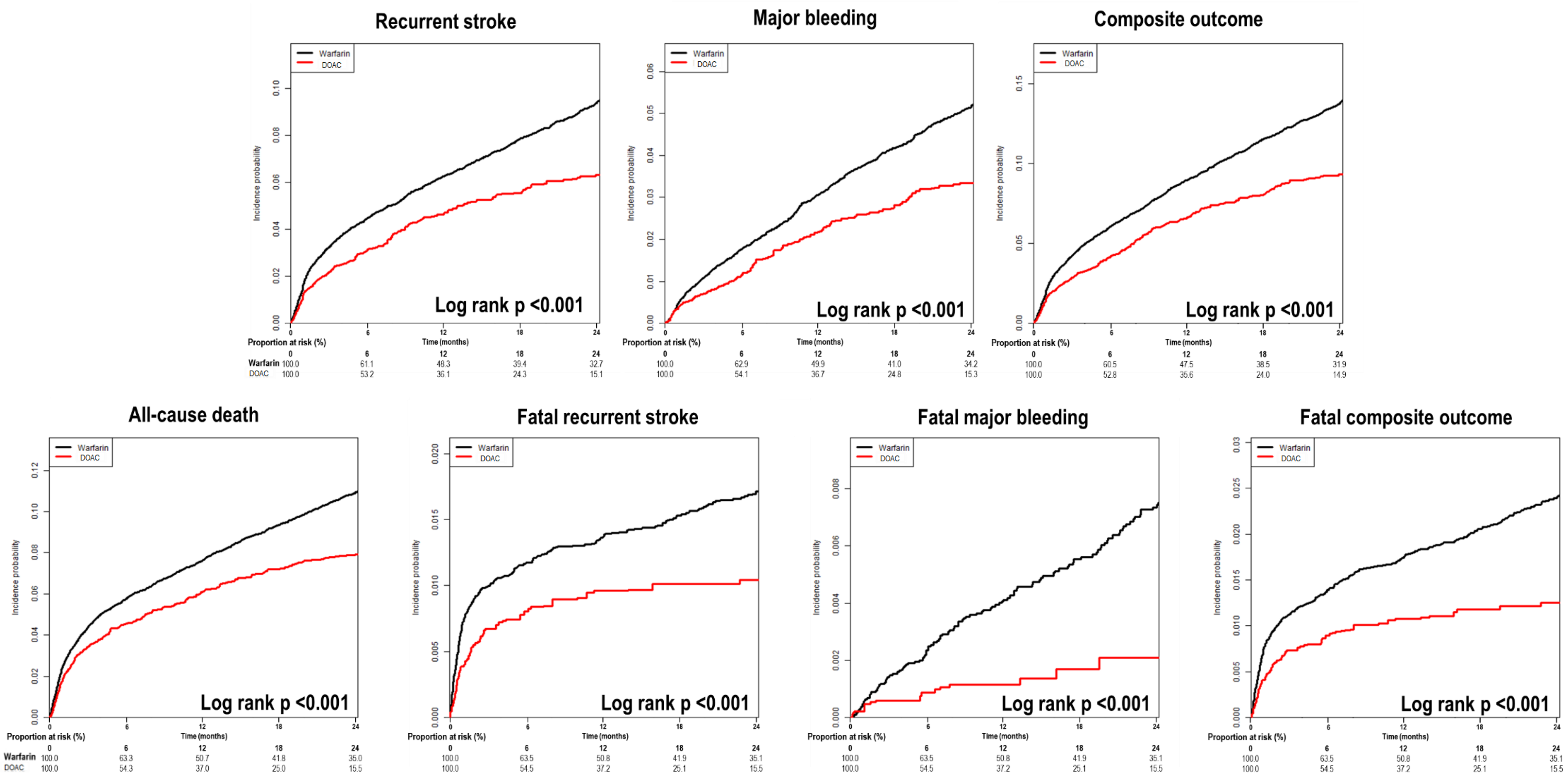

Figure S6. Kaplan-Meier curve of clinical outcomes among patients with warfarin and regular dose DOAC

DOAC = direct oral anticoagulant

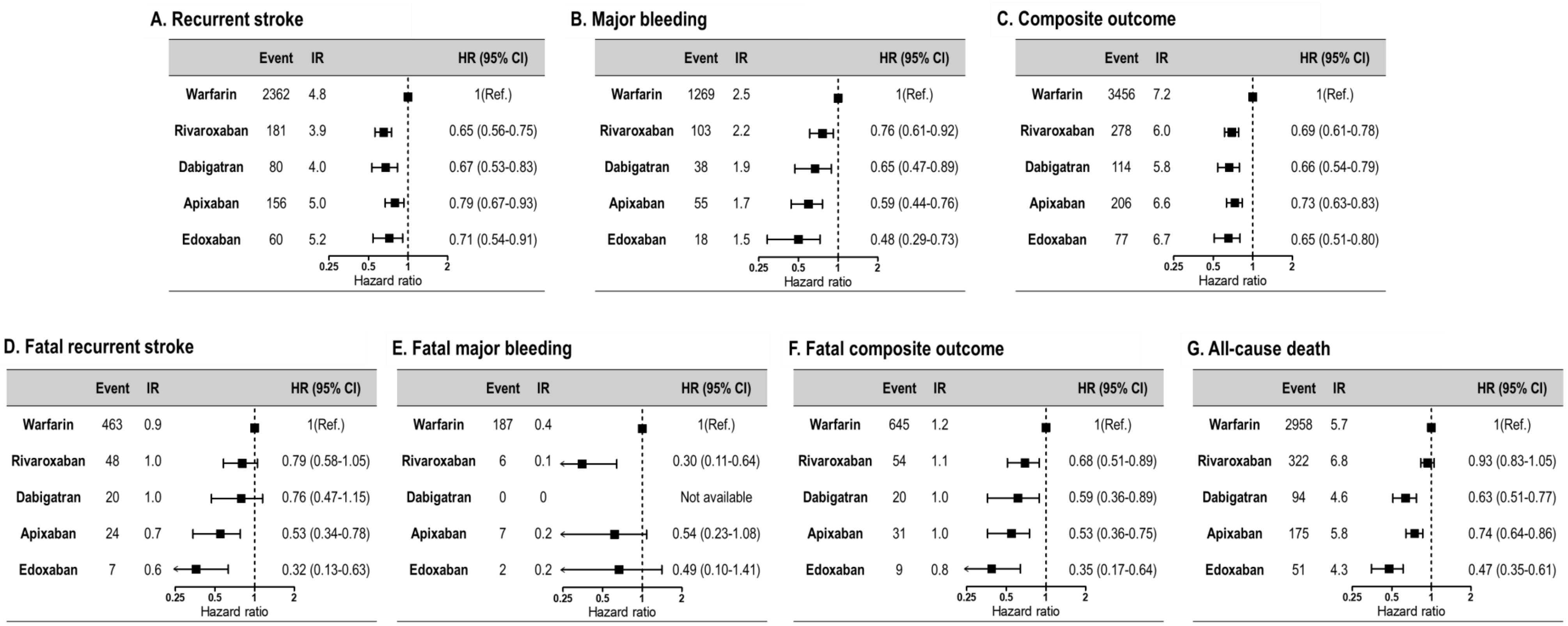

Figure S7. Comparison of clinical outcomes between patients with warfarin and 4 regular dose DOACs

CI = confidence interval, DOAC = direct oral anticoagulant, HR = hazard ratio, IR = incidence rate

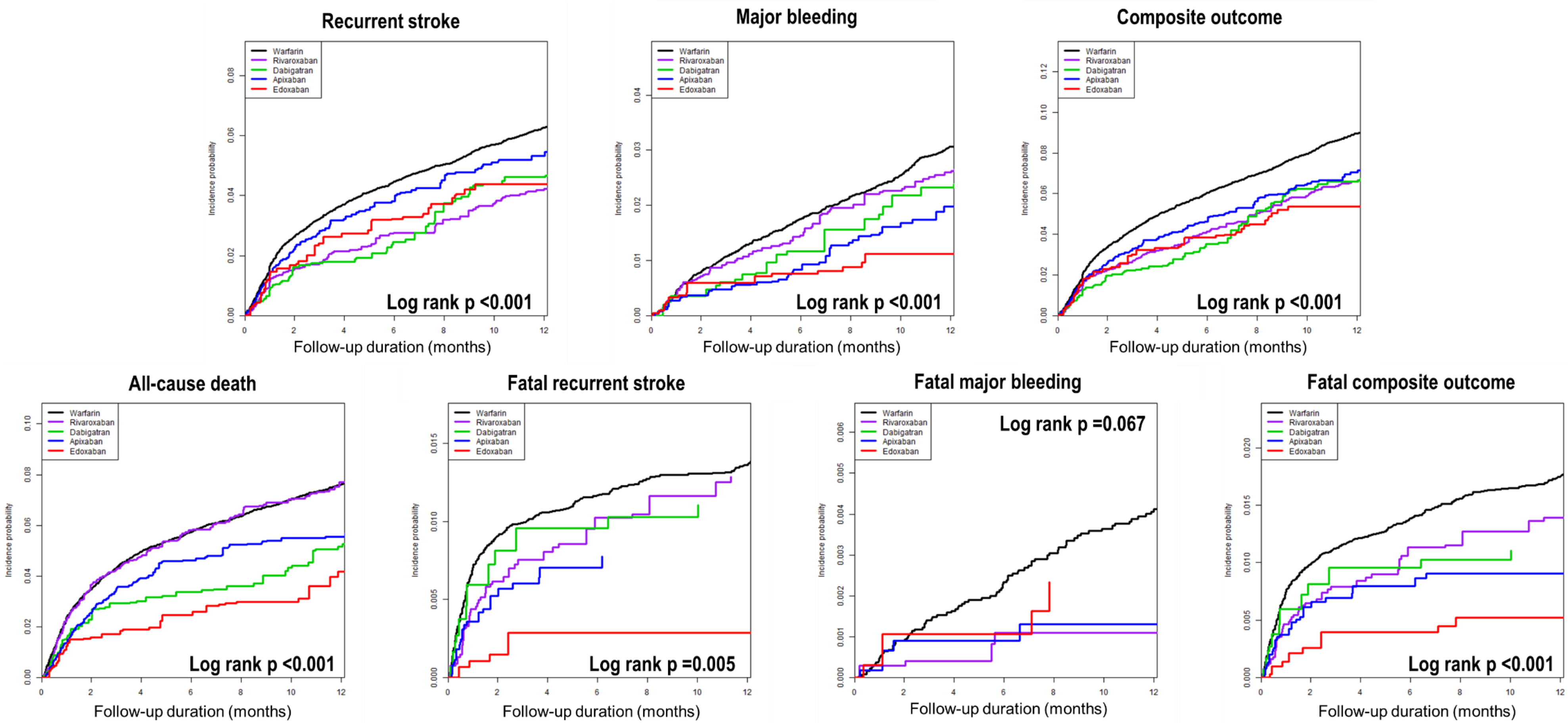

Figure S8. Kaplan-Meier curve of clinical outcomes among patients with warfarin and four different regular dose DOAC

DOAC = direct oral anticoagulant

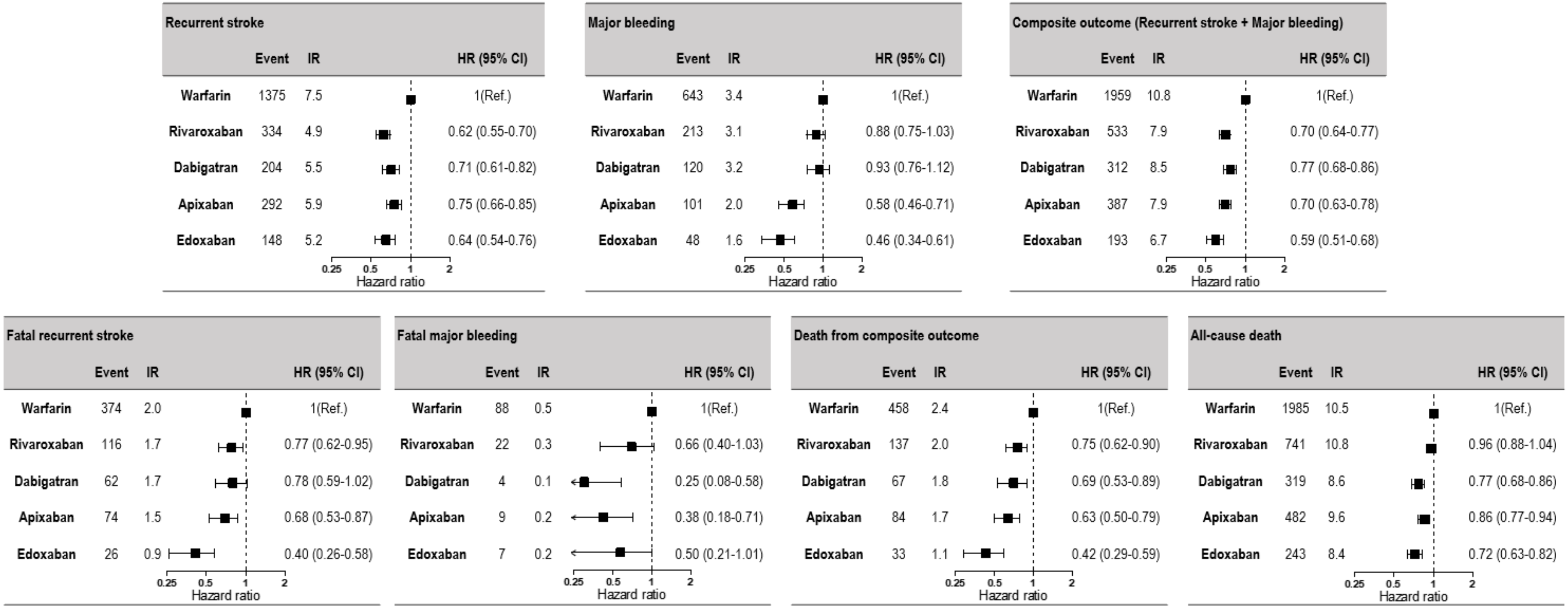

Figure S9. Sensitivity analysis comparing clinical outcomes according to antithrombotic therapy during a 1-year follow-up

CI = confidence interval, HR = hazard ratio, IR = incidence rate
